# Effect of a common *UMOD* variant on kidney function, blood pressure, cognitive and physical function in a community-based cohort of older adults

**DOI:** 10.1038/s41371-021-00608-2

**Published:** 2021-09-30

**Authors:** Linda Elizabeth Villagomez Fuentes, Engi Abdel–Hady Algharably, Sarah Toepfer, Maximilian König, Ilja Demuth, Lars Bertram, Reinhold Kreutz, Juliane Bolbrinker

**Affiliations:** 1 Charité – Universitätsmedizin Berlin, corporate member of Freie Universität Berlin and Humboldt-Universität zu Berlin, Institute of Clinical Pharmacology and Toxicology, Charitéplatz 1, 10117 Berlin, Germany; 2 Charité – Universitätsmedizin Berlin, corporate member of Freie Universität Berlin and Humboldt-Universität zu Berlin, Department of Endocrinology and Metabolism, Augustenburger Platz 1, 13353 Berlin, Germany; 3 Berlin Institute of Health at Charité – Universitätsmedizin Berlin, BIH Center for Regenerative Therapies BCRT, Augustenburger Platz 1, 13353 Berlin, Germany; 4 Lübeck Interdisciplinary Platform for Genome Analytics, Institutes of Neurogenetics and Cardiogenetics, University of Lübeck, Lübeck, Germany; 5 Center for Lifespan Changes in Brain and Cognition, Department of Psychology, University of Oslo, Oslo, Norway

**Keywords:** Cardiovascular diseases, Genetics research, Risk factors, Genetics, Ageing

## Abstract

In genome-wide association studies, genetic variants in the *UMOD* gene associate with kidney function, blood pressure (BP), and hypertension. Elevated BP is linked to kidney function and impaired cognitive as well as physical performance in later life. We investigated the association between *UMOD* rs4293393–A > G and kidney function, BP, cognitive and physical function in the Berlin Aging Study II (BASE–II). Data of 1556 older BASE–II participants (mean age 68.2 ± 3.7 years) were analyzed. BP was determined by standardized automated measurements, estimated glomerular filtration rate (eGFR) by CKD Epidemiology Collaboration creatinine equation. Cognitive function was assessed by Mini-Mental State Examination and Digit Symbol Substitution Test, while physical function by Handgrip Strength and Timed Up and Go-Test. Association analyses were performed by covariance and logistic regression models adjusting for sex. G–allele carriers at *UMOD* rs4293393 exhibited significantly higher eGFR values compared to non–carriers (AA, 76.4 ml/min/1.73 m², CI: 75.7–77.2 vs. AG, 78.4 ml/min/1.73 m², CI: 77.3–79.5 vs. GG, 78.5 ml/min/1.73 m², CI: 75.4–81.7; *P* = 0.010), and a lower risk of eGFR < 60 mL/min/1.73 m^2^ (AG, OR: 0.63, CI: 0.41–0.97, *P* = 0.033). However, *UMOD* rs4293393 genotypes were not associated with BP, diagnosis of hypertension or cognitive and physical function parameters. Our data corroborate previous findings on the association of *UMOD* rs4293393-G with better kidney function in older adults. However, no association between *UMOD* and BP or physical and cognitive parameters in these community-dwelling older adults was detected.

## Introduction

Expression of the kidney–specific protein uromodulin, also known as Tamm–Horsfall protein, is restricted to the thick ascending loop of Henle (TAL) followed by proteolytic cleavage and secretion into the urine [1]. Although it is the most abundant protein secreted in human urine, its functional role is not fully elucidated to date [1]. Uromodulin has been hypothesized to play a role in water and electrolyte balance, urine concentrating ability in the TAL, and in kidney innate immunity [1]. In TAL cells, it can modulate Na-K-2Cl cotransporter (NKCC2) activity and NaCl reabsorption [2].

Common variants in the *UMOD* promoter region such as rs12917707–G > T as well as other single–nucleotide polymorphisms (SNPs) in high linkage disequilibrium (LD) including rs4293393–A > G (complete LD; *r*^2^ = 1.0) have shown genome-wide significant association with estimated glomerular filtration rate (eGFR) and the incidence of chronic kidney disease (CKD) [3, 4]. Moreover, the *UMOD* locus was independently associated with blood pressure (BP) and hypertension [2, 5]. The risk variant of rs4293393, namely the major allele rs4293393–A, was found to increase *UMOD* expression in human kidney samples as well as uromodulin excretion in human urine [2]. Moreover, it was associated with salt–sensitive hypertension and kidney damage in mice and humans [2]. Consequently, by increasing the susceptibility to hypertension and kidney damage during midlife, *UMOD* risk variants may negatively impact cognitive and physical function later in older age. Based on this hypothesis, early identification of individuals at risk might implicitly lead to more effective implementation of preventative lifestyle modifications, optimal BP control and careful follow-up. The age-related increase in BP, particularly systolic BP (SBP), results in a high prevalence of hypertension above 70% among individuals older than 65 years [6–8]. The potential interrelation of BP with cognitive function has received explicit attention from epidemiologic research with mounting evidence supporting their association [9]. According to a recent meta–analysis, persistent elevated SBP ≥ 130 mmHg in midlife, i.e., around the age of 40 years, is an established modifiable risk factor for dementia [10]. Furthermore, lowering BP can reduce the risk of cognitive impairment and dementia [9]. In addition, declines in physical function are accelerated in the hypertensive older adults which further increases their risk for developing functional disability [11] as well as incapacity to perform activities of daily living compared to normotensive older adults [11]. Moreover, reduced lower limb muscle function and muscle mass index as parameters related to sarcopenia, in hypertensive women older than 60 years have been indicated to contribute to lower global cognitive status [12]. As with BP, prevalence of CKD rises with age [13] and cognitive decline is observed in patients with CKD [14, 15]. Studies consistently reported an association between CKD and cognitive impairment [14, 15]. Since it was shown that the *UMOD* rs4293393-G allele has favorable effects on eGFR [3] and diastolic BP (DBP) [2], the associated effect on BP and/or kidney function exerted by *UMOD* rs4293393-G might also contribute to a more favorable cognitive and physical performance status in older adults. To the best of our knowledge, no studies have been conducted to test this hypothesis.

The aim of this study was to investigate the association of an established SNP in the *UMOD* locus, i.e., rs4293393, with kidney function, BP, and comprehensive assessments of cognitive and physical function in participants of the Berlin Aging Study II (BASE–II) aged 60 years and older.

## Methods

### Participants

We performed cross-sectional analyses of baseline data from BASE–II. BASE–II was established as a multidisciplinary and multi–institutional project to study the underlying factors and their interactions that contribute to individual differences associated with aging [16]. Briefly, a population–based cohort including participants living in the greater metropolitan area of Berlin, Germany, was comprehensively investigated from 2009 to 2014. At the time of recruitment, the older subgroup of BASE-II was defined as participants aged 60 years and older. Thus, in this work, we refer to participants of the cohort aged 60 years and older as older adults. To a large extent, participants of BASE-II were originally recruited at the Max-Planck-Institute for Human Development as part of earlier projects with exclusion of subjects with difficulty walking without assistance, a history of Parkinson’s disease, stroke or myocardial infarction, vascular, heart or head surgery, dementia or malignant disease. BASE-II participants have been characterized by a higher education level and an overall better self-reported health status than the general German population (for detailed study description see Bertram et al. [16]). The study was approved by the local Ethics Committee of the Charité–Universitätsmedizin Berlin (EA2/029/09) and all participants gave written informed consent. The BASE–II Steering Committee reviewed our application to use BASE–II data for our analyses and positively voted about the request (19–186).

For the genotype–phenotype association analyses, we used cross-sectional data of the older subgroup and included only those participants with complete genotype information for *UMOD* rs4293393, resulting in an analytical sample of 1556 subjects between 60 and 84 years. Genotypes were derived from Affymetrix SNP Array 6.0 analysis [17].

### Phenotypes

#### Kidney function

Kidney function was determined by eGFR using the CKD Epidemiology Collaboration (CKD–EPI) creatinine equation based on a one–time point measurement of serum creatinine (SCr) [18].

#### BP measurements and definition of hypertension

Attended automated BP measurements in the seated position were performed according to a standard protocol using a validated electronic device (boso–medicus memory, Jung Willingen, Germany) as previously reported [8, 19]. For each participant, two BP measurements for SBP and DBP were performed, one on the right and one on the left arm [8]. The mean of these values was used for statistical analysis and only subjects with complete SBP and DBP measurements were included (*n* = 1529). In a separate analysis, BP was imputed in individuals with antihypertensive treatment by adding 10 and 5 mmHg to the mean SBP and DBP values, respectively [20]. Hypertension was defined as SBP ≥ 140 mmHg and/or DBP ≥ 90 mmHg [6] and/or antihypertensive treatment (self-reported or documented).

#### Assessment of cognitive function, muscle strength, and mobility

The Mini–Mental State Examination (MMSE) served as a tool for screening global cognitive function. It includes tests of orientation, attention, memory, and language, with a maximum score of 30. Scores < 24 are suggestive of cognitive impairment [21]. Attention and processing speed were assessed by conducting the WAIS–II version of the Digit Symbol Substitution Test (DSST) [22].

The Handgrip Strength (HGS) was performed for the measurement of muscle strength and fatigue using a Smedley Dynamometer (Scandidact, Denmark). Three measurements were obtained for each hand and the highest value was used for the current analyses (for details, see [19]). Mobility was assessed by the Timed Up and Go–Test (TUG) measuring the time in seconds for performing gait parameters (stand up, walk, turn, sit down) [19]. A time of 10 s was set as a cut–off value to indicate normal (<10 s) vs. impaired (≥10 s) gait performance [23]. We used both, HGS and TUG, to generally assess the physical function of participants. However, data of questionnaires to measure participants´ physical activity were not available for the current study.

#### Frailty and morbidity

The Frailty index was defined and adjusted according to the definition by Fried. This index is based on five criteria: unintentional weight loss, self-reported exhaustion, weakness, slow walking speed, and loss of physical activity [24]. Dependent on the number of criteria met, participants were classified as frail (3–5 criteria met), pre-frail (1–2), or not frail (no criterion met) [24]. For statistical analysis, frail and pre-frail were combined into one variable.

Morbidity classification was based mainly on the Charlson Comorbidity Index (CCI) [25, 26]. Diagnoses were self-reported or obtained from individual’s reports with selected diagnoses such as diabetes mellitus being verified by an additional laboratory test and computed according to the CCI categories [16]. For statistical analysis, scores ≥ 1 were combined into one variable.

### Statistical analyses

Descriptive data are presented as mean ± standard deviation (SD) or as numbers and percentages. Analysis of covariance was used for genotype–phenotype association analysis in subjects with information on eGFR and SCr values with fixed factor genotype and adjustment for sex as covariate; results are reported as mean and 95% confidence intervals (CI). Data for SBP, DBP, and parameters of cognitive and physical function were analyzed with similar models. Levene’s test for equality of variances was checked and indicated equal variance across compared groups. Binary logistic regression controlling for sex was used to calculate odds ratios (OR) for having eGFR < 60 mL/min/1.73 m^2^, having a TUG result below 10 vs. ≥10 s, being not frail vs. pre-frail or frail, or having a morbidity index score of 0 vs. ≥1. *P* values < 0.05 were considered statistically significant (without adjustment for the number of tests performed). All statistical tests were two-sided and analyses were performed using SPSS 25 (SPSS Statistics Software, Armonk, NY: IBM Corp).

## Results

### Characteristics of the study population

A total of 1556 individuals (51.2% women) with a median age of 68.2 years (range 60–84) were studied. Participants´ characteristics are summarized in Table 1. The morbidity index was 1 or higher in 65% of the participants and about a third of subjects were characterized as pre–frail or frail. Mean eGFR was 77.1 ± 12.1 ml/min/1.73 m^2^ and 139 subjects had eGFR <60 mL/min/1.73 m^2^. Mean SBP and DBP in the seated position were 143.7 ± 18.7 mmHg and 83.1 ± 10.9 mmHg, respectively. Overall, 73% of participants had hypertension.

**Table 1:** Characteristics of the study population.

Parameter	Value
Age (years)	68.2 ± 3.7 (range 60–84)
<65	266
≥6–79	1280
≥80	10
Men	760 (48.8%)
Women	796 (51.2%)
BMI (kg/m^2^), *n* = 1530	26.8 ± 4.2
Waist–to–hip ratio, *n* = 1530	0.96 ± 0.1
Current smoker, *n* = 1542	145 (9.4%)
Morbidity index, *n* = 1419	
0	497 (35%)
≥1	922 (65%)
Frailty index, *n* = 1436	
Not frail	976 (68%)
Pre-frail	448 (31.2%)
Frail	12 (0.8%)
SCr (mg/dl), *n* = 1528	0.90 ± 0.2
eGFR (mL/min/1.73 m^2^)	77.1 ± 12.1
eGFR < 60 mL/min/1.73 m^2^	139 (9.1%)
BP (mmHg), *n* = 1529	
SBP	143.7 ± 18.7
DBP	83.1 ± 10.9
Hypertension	1112 (72.7%)
with antihypertensive treatment	592 (53.2%)
thereof controlled	
BP < 140/90 mmHg BP < 140/80 mmHg	220 (37.2%) 153 (25.8%)
Heart rate (bpm), *n* = 1525	69.5 ± 11.3
MMSE, *n* = 1534	28.5 ± 1.6
DSST, *n* = 1339	44.6 ± 8.5
TUG (seconds), *n* = 1531	7.9 ± 1.9
HGS (kilograms), *n* = 1532	34.2 ± 9.7

Data are given as mean ± standard deviation or as numbers and percentages in parentheses.

*BMI* body mass index, *SCr* serum creatinine, *eGFR* estimated glomerular filtration rate according to CKD–EPI creatinine equation, *BP* blood pressure, *SBP* systolic BP, *DBP* diastolic BP, *bpm* beats per minute, *MMSE* Mini–Mental State Examination, *DSST* Digit Symbol Substitution Test, *TUG* Timed Up and Go-Test, *HGS* Handgrip Strength.

### Analysis of rs4293393 in relation to kidney function, BP, hypertension, and parameters of cognitive and physical function

The minor allele frequency (MAF) of rs4293393 was 18.5% in agreement with current reference data posted on the gnomAD database [v2.1], where MAF in non-Finnish North-Western Europeans is listed with 18.6% [27] and the number (frequencies) of participants with *UMOD* rs4293393 genotypes –AA, –AG, and –GG were 1038 (66.7%), 461 (29.6%), and 57 (3.7%), respectively.

**Fig. 1:**
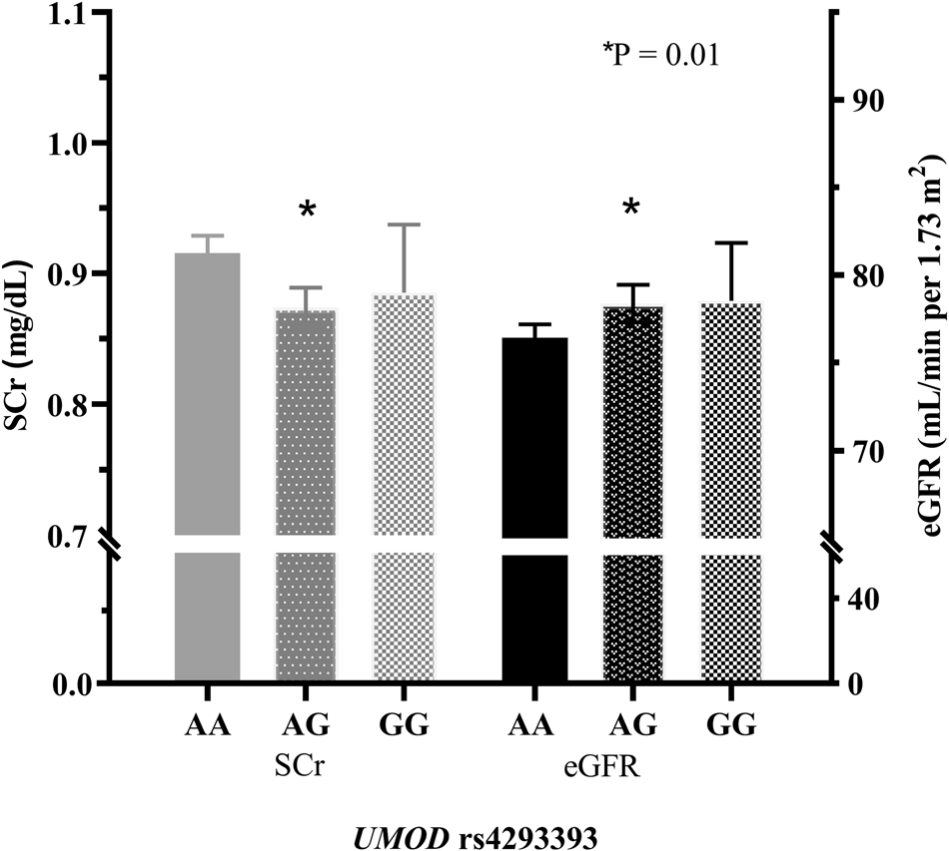
Serum creatinine (SCr) and estimated glomerular filtration rate (eGFR) according to rs4293393 genotypes. *P* = 0.01 comparing mean values of heterozygous AG (*n* = 451) with homozygous AA (*n* = 1021) individuals. Data were analyzed by analysis of covariance.

Carriers of the protective rs4293393 G–allele (based on GWAS by [3, 28]; GG, *n* = 56; AG, *n* = 451) had significantly lower SCr concentrations compared to individuals homozygous for the rs4293393 risk genotype (AA, *n* = 1021, 0.91 mg/dl, CI: 0.90–0.92 vs. AG, 0.87 mg/dl, CI: 0.87–0.90 vs. GG, 0.88 mg/dl, CI: 0.84–0.93; *P* = 0.010, Fig. 1). Accordingly, mean eGFR values were significantly higher in G–allele carriers (AA, 76.4 ml/min/1.73 m², CI: 75.7–77.2 vs. AG, 78.4 ml/min/1.73 m², CI: 77.3–79.5 vs. GG, 78.5 ml/min/1.73 m², CI: 75.4–81.7; *P* = 0.010, Fig. 1). Moreover, heterozygous participants were less likely to have eGFR <60 mL/min/1.73 m^2^ than individuals homozygous (AA) for the rs4293393 risk allele (AG, OR: 0.63, CI: 0.41–0.97, *P* = 0.033; GG, OR: 1.1, CI: 0.45–2.6, *P* = 0.883).

Analyses of covariance adjusted for sex showed no significant association of rs4293393 with mean SBP, DBP, or heart rate (Table 2). We also did not detect an association between rs4293393 and the diagnosis of hypertension as a dichotomous variable (AG, OR: 0.94, CI: 0.73–1.2, *P* = 0.610; GG, OR: 0.83, CI: 0.46–1.5, *P* = 0.532). Further separate analysis for SBP and DBP by adjusting for the use of antihypertensive medication in treated individuals also revealed no significant associations (not shown).

**Table 2:** *UMOD* rs4293393 associations with  BP and heart rate.

Phenotype	*UMOD* rs4293393			*P*
	AA	AG	GG	
BP (mmHg), *n* = 1529	*n* = 1018	*n* = 455	*n* = 56	
SBP	143.3	144.3	145.5	0.519
	(142.2–144.4)	(142.6–146.0)	(140.6–150.4)	
DBP	83.2	83.1	82.0	0.773
	(82.5–84.0)	(82.1–84.1)	(79.2–85.0)	
Heart rate (bpm), *n* = 1525	*n* = 1015	*n* = 454	*n* = 56	
	69.3	70.0	69.3	0.684
	(68.6–70.0)	(68.8–71.0)	(66.4–72.3)	

Data were analyzed by analysis of covariance and are given as means with 95% confidence intervals in brackets adjusted for sex.

*BP* blood pressure, *SBP* systolic blood pressure, *DBP* diastolic blood pressure, *bpm* beats per minute.

Concerning cognitive and physical function, no association of rs4293393 with cognitive function as determined by MMSE and DSST was detected (Table 3), nor with performance in any of the physical function tests including TUG (AG, OR: 0.91, CI: 0.38–2.16, *P* = 0.824; GG, OR: 0.98, CI: 0.69–1.39, *P* = 0.906), HGS (AA, 34.03 kg, CI: 33.7–34.4 vs. AG, 34.63 kg, CI: 34.1–35.2 vs. GG, 33.04 kg, CI: 31.5–34.6; *P* = 0.078), or with morbidity index (AG, OR: 1.05, CI: 0.59–1.89, *P* = 0.858; GG, OR: 1.02, CI: 0.80–1.29, *P* = 0.894) or frailty (AG, OR: 0.87, CI: 0.48–1.59, *P* = 0.655; GG, OR: 0.93, CI: 0.73–1.19, *P* = 0.579).

**Table 3:** *UMOD* rs4293393 associations with cognitive assessments.

Assessment	*UMOD* rs4293393			*P*
	AA	AG	GG	
MMSE, *n* = 1534	*n* = 1024	*n* = 453	*n* = 57	
	28.5	28.5	28.7	0.626
	(28.4–28.6)	(28.4–28.7)	(28.3–29.1)	
DSST, *n* = 1339	*n* = 904	*n* = 391	*n* = 44	
	44.5	44.7	44.3	0.946
	(44.0–45.1)	(43.8–45.5)	(42.0–46.8)	

Data were analyzed by analysis of covariance and are given as means with 95% confidence intervals in brackets adjusted for sex.

*MMSE* Mini–Mental State Examination, *DSST* Digit Symbol Substitution Test.

## Discussion

The common *UMOD* variant rs4293393 minor G–allele was associated with better kidney function in our cohort of community-dwelling older adults in terms of lower SCr concentrations, higher mean eGFR values, and lower odds of having eGFR <60 mL/min/1.73 m^2^. This corroborates previous findings by several genome-wide association studies (GWAS) [4]. These studies unveiled an association between rs4293393 as well as other common *UMOD* variants in LD with SCr [28], eGFRcrea [3, 4], and CKD [3, 4, 28], and are likewise in keeping with our previous work in treated high–risk patients with arterial hypertension with a consistent direction of effect for the minor protective allele [29]. Thus, our current and previous results confirm that *UMOD* variants play an important role in kidney function also in older adults. Yet, this did not translate into associations of rs4293393 with cognitive assessment results in our analysis. Of note, an association between kidney dysfunction based on eGFR and cognitive impairment was reported for eGFR values <60 ml/min/1.73 m^2^ [15]. Although a significant decrease in MMSE values across CKD stages has been reported in frail patients [13], in the Cardiovascular Health Study, in which cognitive function was assessed by the Modified MMSE and DSST, no significant differences were detected between participants with cystatin C-based eGFR ≥ 90 mL/min/1.73 m^2^ and those with eGFR of 60 to below 90 mL/min/1.73 m^2^ at baseline [30]. In addition, in a healthy population aged 50–62 years, a measured GFR below 90 ml/min/1.73 m^2^ but >60 ml/min/1.73 m^2^ did not associate with several tests of cognitive function, including the DSST and MMSE [31]. Moreover, a previous study involving the current cohort did not find an association between mild-to-moderate CKD (Stage G3a; defined as eGFR < 45–59 mL/min/1.73 m^2^) and cognitive performance assessed by MMSE, among other tests [32]. Plausible explanations for our negative findings with respect to the cognitive assessments are the low percentages of participants with eGFR below 60 ml/min/1.73 m^2^ (9.1%) and being frail (0.8%). In general, the prevalence of diseases and age-related comorbidities such as hypertension, heart failure, and diabetes is rather low in our cohort (Supplementary Table S1). Overall, BASE-II participants have a better self-reported health status and a higher educational status compared to the general German population [16]. A better health and higher educational status show an association with reduced risk of cognitive impairment, as does physical activity [33, 34]. Even though a significant association between *UMOD* genotypes and cognitive outcomes could not be detected using the available cross-sectional creatinine measurements, favorable and persisting effects on kidney function during midlife might still affect cognitive status in later adulthood. This should be addressed further in longitudinal studies. Of interest, urinary uromodulin has been suggested as a potential biomarker for cognitive ability in old age [35], and a recent serum analysis in patients with frontotemporal dementia found that serum uromodulin, among other proteins, was dysregulated in patients compared to controls [36]. However, urinary or serum uromodulin data are not available for our cohort.

We could not replicate the association between rs4293393 and BP values or risk of hypertension in our cohort contrary to what has been previously reported in a large GWAS in European individuals where carriers of rs13333226–G displayed a lower risk of hypertension [5]. Yet, and as expected for a complex trait, *UMOD* association with BP values in that GWAS was characterized by fairly small effect sizes of 0.49 mmHg lower SBP and 0.3 mmHg lower DBP per copy of G–allele [5]. Besides its large sample size, an extreme phenotypic definition for both cases and controls was applied in the GWAS: Hypertension cases had SBP ≥ 160 mmHg and DBP ≥ 100 mmHg based on two measurements and were free from any BP lowering medication while controls maintained normal BP over a 10–year follow-up period [5]. Compared to that approach, definition of hypertension was considerably less strict in our analysis corresponding to the definition of hypertension by the European Society of Hypertension [6]. In addition, subjects in our cohort had lower mean BP levels with 143.7 ± 18.7 mmHg for SBP and 83.1 ± 10.9 mmHg for DBP with more than half of the hypertensive participants (53%) treated with antihypertensive drugs (Table 1). In a previous work, we also did not detect a significant association between the *UMOD* variant rs12917707-G > T and 24 h SBP or DBP in a cohort of 1218 patients with hypertension and cardiovascular disease [29]. All of those patients were treated with antihypertensive pharmacotherapy and overall BP was well controlled. Our results are, however, subject to some limitations. As this analysis is based on cross-sectional data of the BASE-II study, parameters like SCr were assessed at a single time point only and other markers of kidney damage were not available for analysis. We thus relied solely on eGFR to delineate kidney function. In addition, and also due to the absence of follow-up data at the time of analysis, we were not able to study potential effects of the investigated genetic variant on long-term changes in BP, cognitive and physical function parameters.

## Conclusions

In summary, our findings confirm and extend previous evidence that genetic variants in *UMOD* associate with kidney function and risk of eGFR <60 mL/min/1.73 m^2^ in community-dwelling older adults, while no association with BP was detected. Importantly, no significant association between rs4293393 and cognitive or physical function were identified in our cross-sectional analysis in a cohort of relatively healthy older adults. Whether or not the association of *UMOD* variants with better kidney function translates into an improved cognitive and/or physical function in older individuals warrants further studies with longitudinal evaluation.

### Summary Table

#### What is known about topic

- Genetic variants in the *UMOD* gene exhibit favorable effects on kidney function, blood pressure, and risk of hypertension.
- Chronic kidney disease and elevated blood pressure are reported to affect cognitive and physical performance in older adults.
- A favorable effect on kidney function and/or blood pressure by *UMOD* variants might translate into a more favorable cognitive and physical function in later life.

#### What this study adds

- We confirm and extend previous evidence of the association of a common *UMOD* variant with better kidney function and lower risk of decreased eGFR < 60 mL/min/1.73 m^2^ in our cross-sectional analysis in older adults..
- However, no association with blood pressure or parameters of cognitive and physical function was detected in this cohort of relatively healthy older adults, which warrants further studies with longitudinal evaluation

## Supplementary information

TABLE S1. Concomitant diseases of the study subjects (n=1556)
