# Supplementary material for: Effect of a common UMOD variant on kidney function, blood pressure, cognitive and physical function in a community-based cohort of older adults
Source: J Hum Hypertens. 2021 Sep 30;36(11):983–8. doi: 10.1038/s41371-021-00608-2 (PMC9649423; doi:10.1038/s41371-021-00608-2)
Supplement: Supplementary file 1 — TABLE S1. Concomitant diseases of the study subjects (n=1556) [file 41371_2021_608_MOESM1_ESM.pdf]

EFFECT OF A COMMON *UMOD* VARIANT ON KIDNEY FUNCTION, BLOOD PRESSURE, COGNITIVE AND PHYSICAL FUNCTION IN A COMMUNITY-BASED COHORT OF OLDER ADULTS

Supplementary information

TABLE S1. Concomitant diseases of the study subjects (n=1556)

**Concomitant diseases** (self-reported or documented)	**Value**
Diabetes mellitus	201 (13%)
Chronic lung disease	64 (4.2%)
Heart failure	302 (19.8%)
Myocardial infarction	30 (2.0%)
Cerebrovascular disease	70 (4.6%)
Peripheral arterial disease	10 (0.7%)
Hemiplegia	1 (0.1%)
Liver disease	259 (16.9%)
Gastric ulcer disease	406 (26.6%)
Cancer	222 (14.5%)
Human immunodeficiency virus infection	5 (0.3%)
Systemic autoimmune diseases	50 (3.3%)

Data are given as numbers and percentages in parentheses.
